# Computational Abstraction

**DOI:** 10.3390/e23020213

**Published:** 2021-02-10

**Authors:** Raymond Turner

**Affiliations:** School of Computer Science and Electronic Engineering, University of Essex, Colchester CO4 3SQ, UK; turnr@essex.ac.uk

**Keywords:** abstraction, computation, representation, specification, Frege

## Abstract

Representation and abstraction are two of the fundamental concepts of computer science. Together they enable “high-level” programming: without abstraction programming would be tied to machine code; without a machine representation, it would be a pure mathematical exercise. Representation begins with an abstract structure and seeks to find a more concrete one. Abstraction does the reverse: it starts with concrete structures and abstracts away. While formal accounts of representation are easy to find, abstraction is a different matter. In this paper, we provide an analysis of data abstraction based upon some contemporary work in the philosophy of mathematics. The paper contains a mathematical account of how Frege’s approach to abstraction may be interpreted, modified, extended and imported into type theory. We argue that representation and abstraction, while mathematical siblings, are philosophically quite different. A case of special interest concerns the abstract/physical interface which houses both the physical representation of abstract structures and the abstraction of physical systems.

## 1. Introduction

This paper is intended to be a contribution to the “philosophy of computer science” [1]. This is a new discipline and uncovering its core philosophical concerns is still in process [2]. However, it appears to raise subject specific ontological, methodological and epistemological questions. What kinds of things are programs? Are they abstract or concrete? There are conceptual questions that arise in connection with the multiple languages of the discipline. What is a “good” language design? What is the function of semantic theory; does it raise parallel issues to those of the philosophy of language? There are methodological and epistemological questions pertaining to the construction of “correct” programs and software. What is the nature of program correctness? Is it a mathematical or empirical affair—or both? Can we guarantee correctness?

The nature of computational abstraction invokes several of these traditional philosophical categories, and is a common theme in the informal arena of computational discussion [3]. It is said to be one of the mechanisms behind the design of programming and specification languages: it is the mechanism that supports the move to “high-level” from machine oriented languages. It is also at the centre of program and software specification and design in the guise of “ data abstraction” [4,5,6,7], and it is this area where we focus attention.

Finding a representation for a given specification is the essence of programming: some goal is specified, and the programming task is to locate a suite of programs that satisfy it. At first gloss, specifications employ abstract concepts that involve little or no process information: specifications say “what has to be done” without saying “how to do it”. Presumably, the latter is the job of actual programs. But this is an over simplification. In practice, “programming” and “specification” are relative terms. In particular, one level of programs can serve as the specification of more concrete ones. For example, Pascal [8] or Miranda [9] may be employed as the specification medium for programs that are eventually implemented in some form of machine code. In these cases, what is the specification and what is the program is a matter of intention: what is taken to provide the correctness conditions for what [10]. What is generally true is that the vehicles of specification, the concepts employed, are at a more “abstract level” than those of the representation or program. Moreover, these different levels of abstraction are enshrined in different notions of data type that are built into contemporary programming and specification languages [4,8,11,12,13,14,15,16]. For instance, Haskell [14] employs recursive data types whereas Fortran [11] uses arrays and iteration. Some data types are “abstractions” of more “concrete” ones in the sense that some information is neglected, hidden or ignored in order to arrive at a more abstract structure. But what is it to neglect, hide or ignore information? What precisely is “computational abstraction”? Is there an exact mechanism for such? Addressing these questions is a crucial part of the present task.

In Section 2, Section 3, Section 4 and Section 5, we introduce our formulation of abstract types, and provide some working examples to illustrate representation and abstraction. Section 6 and Section 7 introduce “Fregean abstraction” in a type-theoretic setting, and Section 8 brings representation into the picture. Section 9, Section 10, Section 11 and Section 12 consider levels of abstraction, abstraction over families and physical abstraction. Section 13, Section 14 and Section 15 discuss the mathematical and philosophical differences between abstraction and representation, and the last section reflects a little on the appropriate foundational framework.

### 1.1. Methodology

As we said at the outset, the present paper is a contribution to this general area. This work is theoretical and foundational: there are philosophical and mathematical aspects to the research. But there is no empirical component; there are no experimental results.

### 1.2. Previous Work

While there is a good amount of contemporary philosophical work aimed at providing a philosophical foundations for abstraction in mathematics [17,18,19,20,21], there is no substantial parallel study aimed at abstraction in computer science. The present paper attempts to fill this gap by applying the insights of this philosophical work aimed at mathematics to computer science. This work has its origins in [2] where the present approach is sketched.

### 1.3. Results

We provide a conceptual and mathematical analysis of “data abstraction” and “representation” and their relationship. In particular, we provide a type-theoretic approach to Fregean abstraction: abstraction generates new abstract types. We demonstrate that abstraction and representation are related to each other as “congruence” and “homomorphism”. However, there is a philosophical twist in that our treatment of abstraction is not based on the normal construction of “Quotient Types”, but on a method of abstraction that has its origins in Frege [22], and underpins contemporary abstractionism in the philosophy of mathematics [17,23,24]. One particular instance of the abstraction/representation pairing sits on the abstract/physical interface, and its analysis provides some insight into physical abstraction/representation, and how this impacts upon any related notion of computation.

## 2. Abstract Data Types

Representation and abstraction come together in the notion of an “abstract data type” [4,7,25], the original formulation of which emphasizes the idea that it is the operations of the type that play the characterizing role.

An abstract data type defines a class of abstract objects which is completely characterized by the operations available on those objects. This means that an abstract data type can be defined by defining the characterizing operations for that type. [7]

According to this, an abstract type is somehow constituted by its collection of operations. There are various interpretations of this [6], but a minimal one has it that an abstract type has the following form. (There is a distinction between “state-based” and “functional” abstract types. We shall concentrate on the latter—which will be sufficient to illustrate the broad idea of abstraction. See [26] for an account of abstraction in state based systems.)

A=<A,Ω>

Here, *A* is the carrier type and Ω is the set of functions that operate on *A*. (One can think of these as set-theoretic structures where *A* is a set and the functions are set theoretic functions. We shall later discuss this assumption in more detail).

But what does it mean to say that a type is completely characterized by “defining” its characterizing functions? There are two kinds of definitions that seem appropriate. An “explicit” definition of a function would be one that defines it in terms of something else, presumably in terms of functions from some other type. For example, we might specify the type of queues by their representation as sequences. So that the queue operations are defined in terms of sequence operations. Subsequently, the properties of the definiendum are fixed by the properties of the definiens. This interpretation is adopted in some approaches to specification [5]. Alternatively, definitions are taken to be “implicit” where an abstract type is fixed by the relationships between these functions expressed mathematically: an “implicit” definition involves an axiomatic account that lays out the formal relationships between the functions of the type. We shall adopt this approach.

However, similar conceptual points arise for both styles. In particular, under both regimes abstract types define abstract objects. This rules out any account where the functions are characterized by some form of physical implementation in which the objects are physical and the operations mechanical operations on these objects. This would not characterize an abstract type. So, to be clear, the characterization we employ follows a traditional philosophical distinction: abstract types are mathematical objects and non-abstract ones refer to physical structures. But be aware that we shall use the terms “abstract” and “concrete” as relative terms. In particular, “concrete types” maybe be mathematical. In contrast, the term “physical type” is taken only to refer to a physical structures. (Eventually within computer science, as opposed to mathematics, all abstract types must be given physical representation. This is in keeping with what we have argued elsewhere: computer science is centrally concerned with the construction of technical artifacts [2]. So abstract types must eventually morph into physical ones).

To make our general notion more applicable, a few extensions and qualifications are necessary. Our structures may take the following form.

A[T]=<A[T],Ω>

This introduces a type that is “polymorphic” in *T*, where the latter is a parameter of the abstract type, i.e., it introduces a whole collection of types parameterized in *T*. Moreover, the functions of the abstract type are implicitly polymorphic, i.e., they maybe predicatively applied to any *T*. Additionally, these structures are subject to some axiomatic constraints.

We shall also require relations as well as functions in our structures. Here we represent them as Boolean valued functions (T⇒Bool), where *T* is a type and the type of Boolean values is taken to be the following structure.

Bool=<Bool,Δ>

Here Bool is the enumerated type {true,false} and cond is the only function in Δ with the following functionality.

cond:Bool⊗T⊗T⇒T

where A⊗B represents the Cartesian product of two types and A⇒B the type of functions from *A* to *B*. Note that Cartesian products are taken to bind more strongly than function spaces, e.g., Bool⊗T⊗T⇒T stands for (Bool⊗T⊗T)⇒T.

The function cond is taken to satisfy the following equality conditions.

$$cond\left(true,t,t^{\prime }\right){=}_{Bool}t\;cond\left(false,t,t^{\prime }\right){=}_{Bool}t^{\prime }.$$

In order to state the axioms, such as these for cond, we assume that every type *T* comes equipped with an “external” notion of equality ${=}_{T}$. For example, ${=}_{Bool}$ is the “external” equality for the Boolean type. Here we shall often abbreviate $b{=}_{Bool}true$ as just, *b* i.e., when used as a logical assertion. Where it is clear which types are involved we shall drop the subscript on equality.

These equalities are not part of the “programming language” but part of its semantic theory [27,28]. You cannot employ them in the text of function definitions, i.e., programs. Note that cond is polymorphic in that it applies to arbitrary types *T*. In particular it applies to Bool itself. So, all the standard logical connectives, ∧,∨,→,¬ may be defined (or “programmed”) in terms of the conditional, e.g.,

$$\to \left(b,b^{\prime }\right)≐cond\left(b,b^{\prime },true\right).$$

More often than not, we shall employ infix notation and write $b\to b^{\prime }$, etc.

In addition, we assume that each type *T* comes equipped with an “internal” Boolean function of equality that is part of the “ functional programming language”.

$$eq_{T}:\left(T⊗T\right)\Rightarrow Bool.$$

That is, we may write function definitions using it. In particular, for complex type constructors, internal equality is usually definable in terms of the internal equality for its components. For example, for Cartesian products, we define it point-wise.

$$eq_{A⊗B}\left(\left(x,y\right),\left(u,v\right)\right)≐eq_{A}\left(x,u\right)\wedge eq_{B}\left(y,v\right).$$

For the types of this paper these two notions of equality will be provably co-extensional. However, both notions are necessary. Semantic judgments about the language are made with the external notion, whereas programs in the language employ the internal one.

This provides the basic notion of “abstract type”. There are some central examples that will be used to illustrate and make the various issues concrete.

## 3. Some Data Types

Lists form a paradigm example of an abstract type. The polymorphic version takes the following form.

L[T]=<L[T],{nil,cons,rec,head,tail}>

For instance, we might form lists of numbers L[N] or lists of Boolean values L[Bool] etc. Formally, the first and last pairs of operations have the following functionalities.

nil:L[T]

cons:T⊗L[T]⇒L[T]

head:L[T]⇒T

tail:L[T]⇒L[T]

The functions nil and cons are the constructors of the type; they dictate the form of the elements. There is an unsaid assumption that the type of lists, over a given type, is the smallest type that contains the empty list nil and is closed under the cons operator. Implicitly, this justifies the use of induction as a means of reasoning about these types. The exact form of induction will depend upon the logical system employed. For example, a formulation in first-order logic would support first-order induction, and this is sufficient for the present application. However, a full development of the underlying theoretical framework is a topic for another occasion, but see the section on foundations for some further elaboration of what this might look like.

The interaction of these constructors with the destructors, head and tail, is governed by the following axioms.

head(cons(t,l))=ttail(cons(t,l))=l

The axioms are silent on what happens outside these constraints. The final operator of the type, the function rec, is a (polymorphic) recursion operator over lists.

rec:L[T]⊗A⇒C

where *A* and *C* are arbitrary types. Given g:A⇒C and f:T⊗A⊗C⇒C, the recursion operator is taken to satisfy the following recursion equations, where the type *A* is an optional parameter, i.e., there may not be one.

rec(nil,a)=g(a)

rec(cons(t,l),a)=f(t,a,rec(l,a))

The function rec enables the definition of new functions, i.e., programming with lists in the functional style is largely driven by the definition of recursive functions.

Our second abstract type is a polymorphic version of *queues*. While queues and lists have the same structural signature,

Q[T]=<Q[T],{emp,enqueue,rec,front,dequeue}>,

lists operate a “last-in” and “first-out” regime: queues reverse matters and employ a “last-in” and “last-out” one. More explicitly, the functions of the structure have the following types.

emp:Q[T]

enqueue:Q[T]⊗T⇒Q[T]

rec:Q[T]⊗A⇒C

dequeue:Q[T]⇒Q[T]

front:Q[T]⇒T

They are are taken to satisfy the following equality axioms. These are a little more messy than those for lists. Notice in particular that dequeue has to recursively unpack the structure of the queue to reach the front.

front(enqueue(emp,t))=tdequeue(enqueue(emp,t))=emp

front(enqueue(q,t))=front(q)whereq≠emp

dequeue(enqueue(q,t))=enqueue(dequeue(q),t)whereq≠emp

Despite these differences, we can employ recursion on queues in a parallel fashion to that for lists. Given g:A⇒C and f:T⊗A⊗C⇒C, recursion over queues satisfies the following.

rec(emp,a)=g(a)

rec(enqueue(q,t),a)=f(t,a,rec(q,a))

We shall also employ restricted abstract types $L{\left[T\right]}^{-}$ and $Q{\left[T\right]}^{-}$ where $\Delta_{L}=\left\{nil,cons,rec\right\}$ and $\Delta_{Q}=\left\{emp,enqueue,rec\right\}.$ All the “programming” in the next section only employs these restricted structures.

## 4. Some Infrastructure

We require some infrastructure to facilitate the formulation of data abstraction, and this is obtained by programming within these structures. (The reader unfamiliar with functional programming might consult [14,15] for a relevant introduction.) Most of the functions or “programs” contained here employ the above form of recursive definition. Indeed, for pedagogical reasons, we shall often not explicitly conform to the exact syntax for recursive definitions but use a more familiar and natural recursive style. We illustrate matters with lists, but everything works just as well for queues.

**Figure 1:**
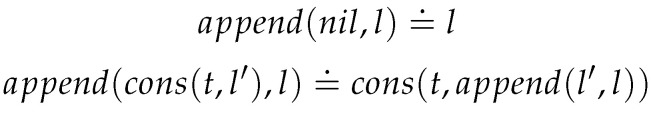
append:L[T]⊗L[T]⇒L[T].

Figure 1 provides a recursive definition of the append operation that glues two lists together.

Here f:T⊗L[T]⇒L[T] is given as f(t,l)=cons(t,l).

**Figure 2:**
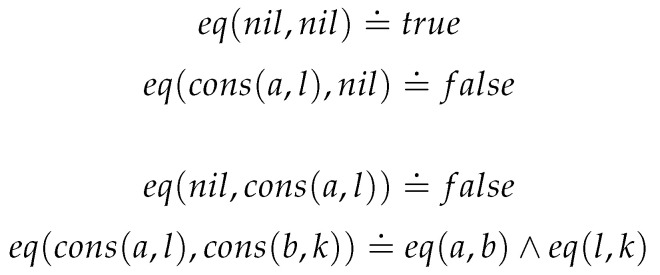
List Equality.

Given internal equality for the type *T*, Figure 2 provides a definition of internal equality for lists.

**Figure 3:**
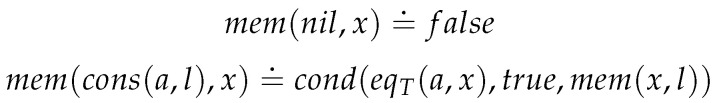
mem:L[T]⊗T⇒Bool.

A few more functions are required for the theoretical development. Membership in lists is defined in Figure 3.

Here mem is defined as a recursive function where the generating function is defined as $f\left(l,a,x,c\right)≐cond(eq_{T}\left(a,x\right),true,c)$. We shall employ more familiar notation and write a∈l for mem(l,a). By definition, the empty list behaves as expected: i.e., as a Boolean valued term x∈nil equals false.

**Figure 4:**
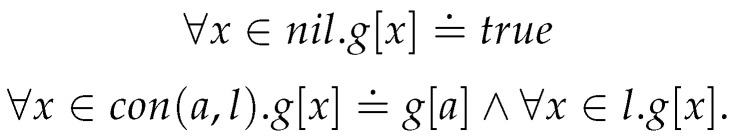
Quantification Over Lists.

Given a Boolean valued function g:L[T]⇒Bool, we may define (Figure 4) “quantification” over lists.

**Figure 5:**
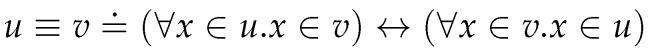
≡:L[T]⊗L[T]⇒Bool.

We may then define “extensional equivalence” for lists, (≡, Figure 5), again as a Boolean valued function.

Finally, we shall say that function g:L[T]⊗A⇒L[T] is Extensional if

∀u:L[T].∀v:L[T].u≡v→∀x:T.g(u,x)≡g(v,x).

We shall use this shortly. All of the above are also definable for queues.

Our objective is to employ these types as a basis for abstracting more abstract ones. In particular, we aim to abstract the following “more abstract” type.

## 5. Finite Sets

The abstract type of finite sets underpins the style of specification common to logical specification languages such as Z [29], B [30] and VDM [5]. Indeed, there is also at least one programming language with finite sets as its central data type [31]. Our objective is to abstract the abstract type of finite sets from lists/queues. For pedagogical reasons, we first put in place this target of abstraction. It has the following signature.

S[T]=<S[T],{ϕ,⊕,rec}>

Here S[T] consists of “finite sets” whose elements are selected from *T*. The type is defined axiomatically as follows. We employ the standard constant for the empty set, and the function ⊕ adds a single element to a set. It satisfies two axiomatic constraints that demand that we ignore duplicates and the order of the elements.

(Dup)⊕(t,s)=⊕(t,⊕(t,s))

$$\left(Ord\right)\;⊕\left(t,⊕\left(t^{\prime },s\right)\right)=⊕\left(t^{\prime },⊕\left(t,s\right)\right)$$

As we shall see shortly, these guarantee extensionality for sets. This paves the way for the formulation of recursion over finite sets. This follows the pattern of recursion for lists. However, it has to be restricted: rec must be a function and, given the above constraints on set equality, for this to hold, we require rec to satisfy the following.

(Duprec)rec(⊕(t,⊕(t,s)),a)=rec(⊕(t,s),a)

$$\left(Ordrec\right)\;rec\left(⊕\left(t,⊕\left(t^{\prime },s\right)\right),a\right)=rec\left(⊕\left(t^{\prime },⊕\left(t,s\right),a\right)\right)$$

Consequently, the generating function f:T⊗A⊗C⇒C must satisfy

(Dupf)f(t,a,f(t,a,c))=f(t,a,c)

$$\left(Ordf\right)\;f\left(t,a,f\left(t^{\prime },a,c\right)\right)=f\left(t^{\prime },a,f\left(t,a,c\right)\right)$$

Such functions we shall call legitimate generators for recursion. *Dupf* and *Ordf* guarantee that recursion is functional. Conversely, if rec is a function that satisfies the recursion equations, then the generating function *f* will be legitimate. Notice that the legitimacy constraint may also be applied to lists, i.e., we can restrict list recursion to legitimate generators.

In particular, the following functions are supported by such recursions.

mem:S[T]⊗T⇒Bool

This is defined exactly as membership in lists. Again, we shall write a∈s for mem(s,a).

**Figure 6:**
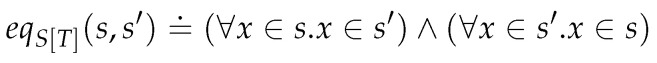
Equality.

We may define quantification with respect to sets in a parallel way to lists. But now we may define internal equality (Figure 6).

Given this, we can use induction on sets, together with *Dup* and *Ord*, to show that extensionality holds, i.e.,

$$\forall x:S\left[T\right].\forall y:S\left[T\right].eq_{S[T]}\left(s,s^{\prime }\right)\to s{=}_{S[T]}s^{\prime }$$

So, “sets” behave as expected. We are now in a position to develop our approach to data abstraction.

## 6. Frege on Abstraction

The word “abstraction” is used throughout computer science with varying degrees of explicitness and precision. Actually, it is doubtful that there is just one computational usage. We concentrate on “data abstraction” [4,5], and here the Fregean perspective seems to provide the beginnings of some conceptual clarification, as well as the basis for some mathematical precision.

The traditional analysis of abstraction has its roots in Locke [21,32]. He has it that abstraction is a mental process in which new abstract ideas are formed by reflecting upon several objects or ideas, and omitting the features that distinguish them.

The same Colour being observed to day in Chalk or Snow, which the Mind yesterday received from Milk, it considers that Appearance alone, makes it representative of all of that kind; and having given it the name Whiteness, it by that sound signifies the same quality wheresoever to be imagin’d or met with; and thus Universals, whether Ideas or Terms, are made. [32]

Seemingly, general terms stand for abstract ideas that are created by separating these ideas from the spatial and temporal qualities of particular things. For instance, one is given a range of white things of varying shape and sizes, and one ignores the respects in which they differ. In this way we come to idea of “whiteness”.

Influential as it has been, it does not provide a clear basis for any precise mathematical account. Fortunately, contemporary work in the foundations of mathematics [17,19,23], based upon Frege’s remarks on mathematical abstraction [22], has laid the groundwork for such an account.

The judgment ’Line a is parallel to line b’, in symbols: a || b, can be taken as an identity. If we do this, we obtain the concept of direction, and say: ’The direction of line a is equal to the direction of line b’. Thus we replace the symbol || by the more generic symbol =, through removing what is specific in the content of the former and dividing it between a and b. We carve up the content in a way different from the original way, and this yields us a new concept. [22]

Frege observes that many of the singular terms that appear to refer to abstract entities are formed by means of functional expressions. For example, the following would appear to pick out new abstract objects, namely *directions* and collections.

- *The direction of a line.*
- *The collection of elements in a list.*

While it is true that many singular terms formed by means of functional expressions denote ordinary concrete objects: e.g., ‘the present pope’, ‘the source of corona 19’, the functional terms that pick out abstract entities are distinctive in the sense that associated with such a functional expression there is an “equation” of the following form.

1. *The direction of a line A = The direction of line B* if and only if *A is parallel to B.*
2. *The collection of elements in list l* = *the collection of elements in list k*

Inspired by these examples, an abstraction principle may be formulated as a bi-conditional of the following form:

∀x:K.∀y:K.h(x)=h(y)↔R(x,y),

where *h* is a term forming operator, *R* is an equivalence relation, and *K* is the “kind” of objects over which we are quantifying and abstracting. Such principles of abstraction are intended to be mechanisms for the abstraction of new “kinds of things”. Given a kind of thing *K*, abstraction introduces a new kind of thing *H* such that:

∀z:H.∃x:K.h(x)=z.

This insists that *h* is a surjective term forming operator from *K* to *H*. So, given the *kind* of thing that are lines, the abstraction principle introduces the *kind* of thing that are directions.

Our goal is to use this insight to provide a mathematical foundation for data abstraction where *kinds* are replaced by abstract data types.

## 7. Abstracting Abstract Types

When applied to data types we require a little more than the above general account of abstraction. More exactly, when dealing with data types we need to explicitly take into account not just the objects but also the functions of the type. Our objective with the new abstract type is to maintain the signature of the concrete type but with a “re-carving” of the content.

The following concept is a modification of the standard notion to cover functions that take and return values (e.g., Bool) outside the type under scrutiny.

**Definition** **1.** *Let A=<A,Ω> be any abstract type then R:A⊗A⇒Bool, an equivalence relation on A, is a CongruenceRelation if every f:Ω,*

*1. If f:B⊗A⊗D⇒A, then $\forall x:B.\forall u,u^{\prime }:A.\forall y:D.R\left(u,u^{\prime }\right)\to R\left(f\left(x,u,y\right),f\left(x,u^{\prime },y\right)\right).$*

*2. If f:B⊗A⊗D⇒C, then $\forall x:B.\forall u,u^{\prime }:A.\forall y:D.R\left(u,u^{\prime }\right)\to f\left(x,u,y\right){=}_{C}f\left(x,u^{\prime },y\right).$*

With this in place we may formulate our notion of data abstraction. Assume that we are given a “concrete” data type

C=<C,Λ>,

together with a congruence relation *R* on C. Then, the following *Principle of Abstraction* is taken to introduce a new type C/R whose elements have the form h(c) for c:C, and whose internal equality conditions are given by the following principle of abstraction.

$$\left(abst\right)\;\forall x:C.\forall y:C.eq_{C/R}\left(h\left(x\right),h\left(y\right)\right)=R\left(x,y\right).$$

In other words, we are postulating a new type via its internal equality. Here the relation *R* is a defined Boolean function of the concrete structure. The new type is introduced whose equality relation is stipulated axiomatically to agree with *R*. Finally, the function *h* is stipulated to be surjective, i.e.,

∀z:C/R.∃x:C.h(x)=z.

This is to guarantee that there are no elements in the abstracted type except those that are abstracted.

Given that *R* is a congruence, we may lift the functions from the concrete type to the abstract one. For example, where for g:Λ with functionality $g:C^{n}\Rightarrow C$, we “lift” the function to $\hat{g}:{\left(C/R\right)}^{n}\Rightarrow C/R$ defined as follows.

$$\hat{g}\left(h\left(x_{1}\right),\ldots ,h\left(x_{n}\right)\right)≐h\left(\hat{g}\left(x_{1},\ldots ,x_{n}\right)\right)$$

This yields a new abstract type.

C/R=<C/R,Λ/R>

where Λ/R is the type of all such $\hat{g}.$

We illustrate the idea by abstracting finite sets from lists. We shall deal with the case of queues, and how matters are related, later.

**Example** **1.** *We first provide the “axis of abstraction”. This is provided by the notion of “extensional equivalence” for lists. The following principle of abstraction generates a new type L[T]/≡ (written as S[T]).*

$$\forall u:L\left[T\right].\forall v:L\left[T\right].eq_{S[T]}\left(set\left(u\right),set\left(v\right)\right)=u\equiv v$$

*This provides the actual carrier of the type. But now we have to define the various operations following the pattern of the general case.*

ϕ≐set(nil)

a⊕set(l)≐set(cons(a,l))

rec(set(z),a)≐set(rec(z,a))

*where both set and list recursion are restricted. Each of these new operators is extensional, i.e., congruent relative to extensional equality.*

We have thus extracted a structure that satisfies the axioms for sets: the conditions Dup and Ord follow since equality is extensional equivalence. To complete the process of abstraction we can “kick away” the dependence on lists and axiomatize finite sets directly in terms of *Dup* and *Ord*.

We might be tempted to interpret these “Fregean abstract types” as quotient types: the new type A/R would then be identified as the set of equivalence classes of *A* induced by the congruence relation *R*. This would be the standard mathematical approach. But this interpretation is not the Fregean one. It does not have the same ontological force. Fregean abstractions are not new sets: principles of abstraction in the Fregean mold introduce new sui-generis notions that are not part of the existing background ontology. Fregean abstraction principles are taken to provide a mechanism for the creation of new abstract structures. Indeed, abstraction in this guise provides an explanation of how new abstract structures might come about.

## 8. Representation

On the face of it, representation is the opposite of abstraction. Here one begins with an abstract type and seeks to represent it in a more concrete one [4,5]. More precisely, given an abstract type

A=<A,Ω>,

the objective is to locate a more concrete one,

C=<C,Λ>.

This is taken to “represent” the abstract one just in case there exits a function,

F:C⇒A,

the “Representation Function”, from *C* into *A* that reflects the structural connections between them.

**Definition** **2.** *Let C=<C,Λ> and A=<A,Ω> be abstract types, then F:C⇒A, an is a Homomorphism if every $f_{A}:\Lambda$*

*1. If $f_{C}:B⊗C⊗D\Rightarrow C,$ then $\forall x:B.\forall u:C.\forall y:D.(F\left(f_{C}\left(x,u,y\right)\right){=}_{A}f_{A}\left(x,F\left(u\right),y\right)).$*

*2. If $f_{C}:B⊗C⊗D\Rightarrow E,$ then $\forall x:B.\forall u:C.\forall y:D.f_{A}\left(x,F\left(u\right),y\right){=}_{E}f_{C}\left(x,u,y\right).$*

One further demand is that homomorphisms that are taken to be representational mappings are surjective, i.e., all abstract entities must have a concrete representation. So, a representation function must be a surjective homomorphism from the structure C onto the structure A.

**Example** **2.** *In the representation of sets as lists we are required to locate a representing homomorphism:*

set:L[T]⇒S[T].

*And this is straightforward: we follow the structure of lists.*

set(nil)≐ϕ

set(cons(t,l))≐t⊕set(l)

*This is surjective: to prove it we employ the set-induction. So everything in S[T] has the form set(x) for some x:L[T]. Let*

f:L[T]⊗A⇒C

*be extensional. Then we may extend this to a function*

$$\hat{f}:S\left[T\right]⊗A\Rightarrow C$$

*by*

$$\hat{f}\left(set\left(x\right),a\right)≐f\left(x,a\right)$$

*We can prove for restricted recursion for lists, this time by list-induction, that for all z:L[T],*

set(rec(z,a))=rec(set(z),a),

*where on the right hand side $\hat{f}$ is employed in the definition of rec. So, as expected, set extends to the recursion operator.*

There is another notion of representation that refers to the relation between a physical system and the external physical world. This is a case of the physical system “modelling” the physical world. Our notion of representation exists between a physical system and an abstract one. However, the word “implementation” is often used for both notions of representation.

## 9. Programming and Computation

**Figure 7:**
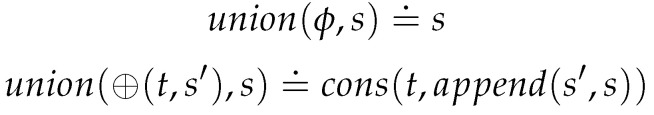
union:S[T]⊗S[T]⇒S[T].

What is a “program” and what is a “computation” differ at different levels of abstraction. Sets are more abstract than lists and this impacts upon the style of programming, and what computations are supported. Programming with sets must respect extensionality. An example of this is the append program for lists. This gives rise to the union program for sets (Figure 7).

The function append is a representation of union where the two are related under the set function extended to set recursion. Conversely, given *append* we may abstract or lift the function to obtain the union operation on sets. This is possible because append is an instance of restricted recursion. So, computations generated by these two functions mirror each other. From the representational perspective, sets fix the specifiable computations on lists; with abstraction only certain list computations give rise to set theoretic ones.

**Figure 8:**
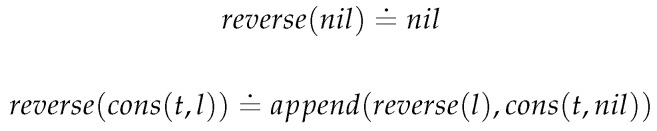
Reversing a List.

However, although the following, Figure 8, is programmable with lists, it is not with sets: it is not a restricted recursion.

So, there are computations with lists that cannot be carried out with sets, and these involve non-extensional functions. If we move down one level to the representation of lists in store in the form of linked lists, there will be computations on linked lists (e.g., those involving pointers to locations) that have no analogues with the abstract notion of list: not all physical computations will have abstract analogues. Different levels of abstraction support different notions of computation. Any computation programmable at a more abstract level is representable lower down but not visa-versa. This brings us nicely to the following section.

## 10. Levels of Abstraction

The computer science literature contains a great many informal discussions of the phrase, “level of abstraction” including its wider philosophical application [33]. There is also a pioneering formal analysis on the identity of computational artifacts [34]. However, there are few if any, conceptually motivated formal accounts of such levels within the literature on data abstraction. Our objective is to provide one based upon the above concept of “Fregean abstraction”.

Any approach to abstraction in the Fregean mold faces the threat of paradox [19,20]. The neo-Fregeans Hale and Wright [23] respond by severely restricting the class of acceptable abstraction principles. Their approach is “static” in the sense that they hold the domain of the overall theory fixed. The alternative approach is “dynamic” [35] in the sense that abstraction with respect to a given domain may result in a new domain: i.e., we do not work in some fixed formal system but allow systems to grow by abstraction, and by abstractions built upon previous abstractions. This is the present approach. (Formally, we expect the addition of new abstract types to be conservative but we need a more precise formal framework to state and prove this).

We illustrate matters with the move from list to sets that hides various possible levels of abstraction. Consider the principles Dup and Ord. These two principles are associated with two intermediate levels of abstraction: one jettisons order and the other eliminates duplicates. Consider the second. The following Boolean valued function, Figure 9, insists that two lists *u* and *v* are permutations of each other.

Here delete(y,u) deletes *y* from *u*. This facilitates the following principle of abstraction: two lists are equivalent if they are permutations of each other. Let u,v:L[T] then

$$\forall u:L\left[T\right].\forall v:L\left[T\right].(eq_{B[T]}\left(bag\left(u\right),bag\left(v\right)\right)=perm\left(u,v\right)).$$

As indicated by the name, this introduces Bags as a new abstract type which we symbolize as follows.

B[T]=<B[T],empty,plus,rec>

Bags are permutation independent (Ord). Recursion is now restricted to generating functions that are order-independent, i.e., satisfy Ordf.

Conversely, given both types, L[T] and B[T], the representation function is then given as follows.

bag:L[T]⇒B[T].

This follows the same pattern as the representation function for sets.

bag(empty)≐ϕ

bag(cons(t,l))≐plus(t,bag(l))

rec(bag(z),a)≐bag(rec(z,a))

This type, which in terms “levels of abstraction”, is intermediate between lists and sets.

Alternatively, beginning with lists, we can abstract by removing duplicates. To this end we define a function, Figure 10, that eliminates duplicates.

We are then able to set up an equivalence between lists.

l≍k≐duplicate(l)=duplicate(k)

Finally, we abstract on this relation.

$$\forall u:L\left[T\right].\forall v:L\left[T\right].(eq_{N[T]}\left(nd\left(u\right),nd\left(v\right)\right)=u≍v)$$

This introduces a new type—lists without duplicates.

N[T]=<N[T],none,ndcons,rec>

Here recursion is restricted to generating functions that satisfy (Dupf).

These two abstractions can be applied not just to lists but to the result of each other: they maybe composed in any order. Either way, the result of the composition is a type whose elements satisfy both Ord and Dup, i.e., this gets us to S[T] by two possible routes.

We can put matters as follows.

**Definition** **3.** *Let C=<C,Λ> and A=<A,Ω> be abstract types. Then A is at Least as* Abstract *than C if there is a surjective homomorphism from C to A. It is* *Strictly Less Abstract* *if in addition there is no homomorphism in the opposite direction.*

*For example, we have surjective homomorphism operating between the following abstract types—and none in the other directions.*

$$L{\left[T\right]}^{-}\Rightarrow B\left[T\right]\Rightarrow S\left[T\right]$$

*Abstraction is a layered dynamic process, and the above examples, demonstrate, in a precise way, how different levels can be formulated and combined under composition to yield even more abstract notions.*

## 11. Abstraction over Families

There are many possible representations of finite set theory: lists, queues, bags, arrays, and sequences all provide such. Moreover, each of these possible representations can be used as the source of abstraction. Indeed, the various mechanisms of abstraction are somehow an encoding of what all these representations have in common. Additionally, what they all have in common is that they are “containers” whose central property is to hold elements.

More generally, how do we abstract a type from a family of abstract types that share some “common” features? To address this we require the notion of a family of abstract types.

**Definition** **4.** *An I-Indexed Family of Abstract Typesis a family*

$$F=<A_{i},\Delta_{i}{>}_{i:I}$$

*where each type has the same functional signature. A simple example employs our existing types.*

**Example** **3.** *Consider the abstract types $L{\left[T\right]}^{-},B\left[T\right]$ and $Q{\left[T\right]}^{-}$ where $\Delta_{L}=\left\{nil,cons,rec\right\},$$\Delta_{Q}=\left\{emp,enqueue,rec\right\},$$\Delta_{B}=\left\{empty,plus,rec\right\}.$ These provide the constituents of a simple indexed family*

$$C=<C_{i},\Delta_{i}{>}_{i:I}$$

*with three components indexed by the enumerated type I={L,B,Q} and where $C_{B}=B\left[T\right]$, $C_{Q}=Q{\left[T\right]}^{-}$ and $C_{L}=L{\left[T\right]}^{-}$.*

*To abstract over such a family we need to be able to articulate what they have in common, and this is achieved by isolating the appropriate notion of congruence for such families. For this it is technically convenient to represent them as single structure over which congruence relations can be formulated in the standard way. Consequently, we form the disjoint union of the types in the family.*

**Definition** **5.** *The* Disjoint Union *of the family $F=<A_{i},\Delta_{i}{>}_{i:I}$ is the structure*

A=(A,Δ)

*where*

*(i)*  $a:A↔\exists i:I.\exists z:A_{i}.a=\left(i,z\right)$
*(ii)*  $f:\Delta ↔\exists i:I.\exists g:\Delta_{i}.\forall x_{1}:A_{i}\cdots \cdots \forall x_{n}:A_{i}.f\left(\left(i,x_{1}\right),\cdots ,\left(i,x_{n}\right)\right)=g\left(x_{1},\cdots ,x_{n}\right)$

**Example** **4.** *The disjoint union of the family of abstract types*

$$C=<C_{i},\Delta_{i}{>}_{i:I}$$

*is the abstract type, “container”.*

C[T]≐(C[T],Δ)

*Explicitly, Δ={nil,cons,rec} where, according to the definition, these are defined as follows. We illustrate with* cons.

cons(t,(L,l))≐cons(t,l)

cons(t,(B,b))≐plus(t,b)

cons(t,(Q,q))≐enqueue(t,q)

*While these form a family with just three members, there are other possible “container” types such as* Sequences *and* Arrays, *and this analysis can be extended in a straightforward way.*

*Let $F=<A_{i},\Delta_{i}{>}_{i:I}$ be any indexed family. The representations of a structure B given in terms of individual members of the family, i.e.,*

$$h_{i}:A_{i}\Rightarrow B$$

*maybe turned into a representation of B in terms of the disjoint union in the obvious way.*

$$h\left(i,a\right)≐h_{i}\left(a\right)$$

*So, in particular, we get a representation of C[T] into S[T].*

We are now in a position to define abstraction over such families.

**Definition** **6.** *Abstraction over Families. Let*

A=(A,Δ)

*be a disjoint union of the family $F=<A_{i},\Delta_{i}{>}_{i:I}$ and let R:A⊗A⇒Bool be a congruence relation over A. Abstraction introduces a new type*

A/R=<A/R,Δ/R>

*with equality given by the following abstraction principle.*

$$\forall u:A.\forall v:A.eq_{A/R}\left(\left[i,a\right],\left[j,b\right]\right)=R\left(\left(i,a\right),\left(j,b\right)\right)$$

*where the new terms take the form [i,a]. Given R is a congruence we may lift the functions of the individual types to A as standard, i.e., Δ/R is the set*

$$\left\{\hat{f}.f:\Delta \right\}$$

*where*

$$\hat{f}\left(\left[\left(i,a_{1}\right)\right],\ldots ,\left[\left(i,a_{m}\right)\right]\right)≐\left[f\left(\left(i,a_{1}\right),\ldots ,\left(i,a_{m}\right)\right)\right].$$

**Example** **5.** *As an example we demonstrate how to abstract sets from containers. Using recursion on the structures we are able to define a congruence relation over C[T]. We illustrate with that part of the relation that links lists and bags (Figure 11).*

*The definitions of the other pairs follow suite: together they define extensional equivalence between the types.*

How is this related to abstraction relative to a single structure? The congruence relation R:A⊗A⇒Bool has restrictions to pairs of types in the family.

$$R_{ij}:A_{i}⊗A_{j}\Rightarrow Bool$$

$$R_{ij}\left(a,b\right)≐R\left(\left(i,a\right),\left(j,b\right)\right)$$

In particular, consider the special case $R_{i}≐R_{ii}$, and the corresponding restricted abstraction:

$$\forall u:A_{i}.\forall v:A_{i}.eq_{A_{i}/R_{i}}\left(\left[i,a\right],\left[i,b\right]\right)=R_{i}\left(a,b\right).$$

Here the congruence is restricted to the elements of a single abstract type. This yields a new abstract type:

$$A_{i}/R_{i}=<A_{i}/R_{i},\Delta_{i}/R_{i}>.$$

**Example** **6.** *In the case of our running example, the abstracted type is isomorphic (≃) to what is obtained from abstraction over a single relation i.e.,*

$$\left(C\left[T\right]/E\right)≃\left(L{\left[T\right]}^{-}/\equiv \right)≃\left(B\left[T\right]/\equiv \right)≃\left(Q{\left[T\right]}^{-}/\equiv \right)≃S\left[T\right]$$

*So, abstracting on a family of containers results in the same type (up to isomorphism) as abstracting from a single member.*

In fact, generally, such single abstractions are isomorphic to the family abstraction.

**Theorem** **1.** *For each i:I,$A/R≃A_{i}/R_{i}$.*

This justifies the use of our original form of abstraction where a new type is abstracted from an individual type.

## 12. Abstract and Physical

We have used the term “abstract” in an ambiguous way. Firstly, we have used the term in a relative sense as a member of an abstract/concrete pair, i.e., one member of a pair is more “abstract” than another. But there is also the absolute sense of the term in which a type is mathematical. So far all types have in this sense been “absolutely” abstract. And this applies to all those we have referred to as “concrete”. Of course, in computer science, at some point in the levels of representation/abstraction, the notion of being “concrete” will give way to the “physical” [36], where a physical type refers to physical things, and as such it is not determined by mathematical means but by the physical properties of its objects and operations. In other words, a physical type

P=<P,Π>,

has physical objects and operations. A simple example concerns digital circuits. Such circuits are not mathematical objects but physical devices. Instances include binary adders, *and* and *or* gates, etc. Complex circuits result from composing simpler ones.

While there are significant differences that emanate from the different natures of abstract and physical devices, the representational/abstraction distinction is still applicable. What then becomes of the processes of representation and abstraction?

Given an abstract type

A=<A,Ω>

and a physical one

P=<P,Π>,

P will be taken to be a representation of A just in case there is a surjective mapping, a correspondence, from the physical device to the abstract one that preserves the structure. This mirrors the demands of the so called *simple mapping* account of computation [37] where each pair of functions in the concrete and abstract types are subject to the constraints of that account. This is reflected in the homomorphic nature of the mapping.

Physical representation may be understood in terms of the concepts of technical artifacts [38,39,40,41]. Technical artefacts are physical objects. However, they cannot be definitively characterized by enumerating their physical properties since this has no place for their functional features. Likewise, they cannot they be completely characterized by an intentional conceptualization: their functionality must be realized in an adequate physical structure. Any adequate conceptualization must take into account both aspects and involve both functional and structural features. See [2,38,39,40,41,42,43] for further discussion of this perspective. Applied to the present setting, the abstract type can be taken as the “functional description of the artifact” and informs us “what the thing is for”. The physical type is characterized by a physical description of its objects and operations and acts as the “structural description” of the artifact.

Unfortunately, we inherit all the philosophical concerns of the simple mapping account of the nature of physical computation [44]. In particular, the homomorphism constraint is taken to be too easy to satisfy: almost anything that can be put into correspondence with the abstract type counts as a representation. Moreover, these triviality results apply also to the abstract/concrete relation (that is defined in terms of homomorphism), namely, that every concrete data type is a representation of every abstract one.

However, there is more going on here than mere extensional agreement that is witnessed by the homomorphism. In particular, this is not the only relationship between physical and abstract devices. Abstracting the abstract device from concrete instances requires the uncovering of a congruence relation. Given a collection of physical devices

P

and a congruence relation *R* on P, we can abstract to obtain an abstract type

P/R.

This yields an abstract device whose mathematical properties are inherited from the structural properties of the various physical devices, i.e., those that survive the congruence. At this point what is a physical property is abstracted as a mathematical one. This is line with the Wittgenstein perspective that mathematical theorems are ‘hardened’ empirical regularities, upon which the former are supervenient. A mathematical ‘proposition’ functions as if it were an empirical proposition “hardened into a rule” [45]. Such abstractions provide one mechanism for transforming empirical propositions to mathematical ones. Once more, we illustrate with digital circuits. We say that two physical circuits *P* and *Q* are “equivalent”, if they have the same input/output. They may differ in terms of their size and the other physical properties possessed by electronic circuits. When we abstract on this relation we obtain a data type of “abstract circuits”.

The representation/abstraction duality is still present in the physical set-up. In the representational case, the use of the concepts of technical artifacts to conceptualize matters seems appropriate. Not so in the case of abstraction. Abstracting from a collection of physical devices yields an abstract characterization of them. Logically, this creates a new concept; it is not artifact specification [10] but the creation of an abstract notion.

We shall return to the abstract/physical case when we have made some more general observations about the differences between representation and abstraction. However, the general point is clear. There is more to the relationship between the physical and abstract devices than their extensional agreement: there is also an intentional aspect that manifest itself as one of either representation or abstraction. This takes some of the sting out of the “pancomputationalism [37]” claim.

## 13. Mathematical Duality

We earlier alluded to the relationship between “abstraction” and “representation”. In particular, we stated that the two are related via two standard mathematical notions (“congruence” and “homomorphism”). This provides us with a way of conceptualizing the connections between representation and abstraction, and ensures us that we have a complementary pair of notions.

The abstraction process itself begins with a congruence relation and implictly defines a homomorphism via the principle of abstraction. The converse is also true: every homomorphism gives rise to a congruence. The following is routine to check.

**Theorem** **2.** *Given types $A=<A,\Omega_{A}>$ and $B=<B,\Omega_{B}>$ with the same signature, and a surjective homomorhism, F:A⇒B we can construct a congruence relation by*

R(x,y)≐(F(x)=F(y))

*Then A/R=<A/R,Ω/R> is isomorphic to B.*

So formally, A is an abstraction of C if and only if C is a representation of A. This is the standard mathematical relationship between homomorphisms and equivalence/congruence relations. Moreover, there are parallel mathematical obligations. In the representation scenario we have to show that the specified mapping is a surjective homomorphism; in the abstraction case we have to show that the defined relation is a congruence. They are mathematical siblings: if one is true so is the other. However, as we remarked before, Fregean abstractions do not result in quotient types but introduce new concepts and structures that are sui-generis.

But even given the mathematical confluence between the two, there is much more to the relationship between concrete and abstract structures than this mathematical one. We shall get to this shortly but first we need to explore the process of abstraction a little more.

## 14. Fully Abstract Structures

Are there abstract types that are, in some sense, as abstract as possible. One motivation for this is pragmatic: a desideratum for specification and design is to isolate and hide as many implementation details as possible. Another is located in a more austere interpretation of the notion of abstract data type. Being fixed by the functions of the type could be taken as a demand that the elements of the type are “indiscernible” with respect to the functions of the type, i.e., they fix the notion of equality for the type. To proceed we require following notion.

**Definition** **7.** *A type B=<B,Ω> is a* Fully Abstract Structure *of a class C of types, of the same signature, if for any A in C there exists a surjective homomorphism from A onto B.*

**Example** **7.** *Consider the class $\{L{\left[T\right]}^{-},B\left[T\right],S\left[T\right]$}. S[T] is the most abstract.*

Given this “Galois” style connection between our notions of congruence and homomorphism, we can also characterize fully abstract structures in terms of congruence relations.

**Theorem** **3.** *A structure B is a fully abstract type of the class C iff for any A in the class there exists a congruence R on A such that A/R is isomorphic to B.*

**Proof.**  Assume that B is fully abstract for the class C. Assume that A is a member of C. Then there is a homomorphism *H* from A into B. Define the derived congruence as before:

a≡b≐H(a)=H(b).

We know from a previous theorem that, A/R is isomorphic to B. Conversely, assume A is in the class and there exists a congruence *R* on A such that A/R is isomorphic to B. Let *G* be this isomorphism. Abstraction itself provides a surjective homomorphism *F* from A onto A/R. The composition G∘F yields the required homomorphism from A onto B.  □

But how do we construct such fully abstract types in a uniform way? This brings the second motivation into the picture. In the next definition we assume that equality $\left({=}_{A}\right)$ for the structure A=<A,Ω> is not an element of Ω.

**Definition** **8.** *Let A=<A,Ω> be any type. Then for each O:Ω we define*

$$a\approx_{O}a^{\prime }≐I\left(a,a^{\prime }\right)\wedge O\left(a,a^{\prime }\right)$$

*where*

$$I\left(a,a^{\prime }\right)≐\forall z:A.O\left(a,z\right)↔O\left(a^{\prime },z\right)$$

$$O\left(a,a^{\prime }\right)≐\forall z:A.O\left(z,a\right)↔O\left(z,a^{\prime }\right)$$

*The first insists that they are indistinguishable as inputs, and the second they are so as outputs. So, $a\approx_{O}a^{\prime }$ insists that a and $a^{\prime }$ are indistinguishable relative to the operation O. The* Natural Congruence *for A, $\approx_{A},$ is then defined as:*

$$a\approx_{A}a^{\prime }≐\forall O:\Omega .a\approx_{O}a^{\prime },$$

*which demands that a and $a^{\prime }$ are indistinguishable relative to the whole structure.*

For following we have only to observe that any congruence will by definition imply the natural one.

**Theorem** **4.** *Let A=<A,Ω> be any type. Then*

*(1) $\approx_{A}$ is a congruence relation on A*

*(2) If R is any congruence on A, then $R\left(a,b\right)\to a\approx_{A}b$*

We may now construct the corresponding abstract type.

**Definition** **9.** *Let A be any state-based structure. Then*

$$A/\approx_{A}$$

*is the Natural Abstraction of A.*

In what sense is the natural abstraction fully abstract?

**Theorem** **5.** *Let A=<A,Ω> be any type. Let $C_{A}$ be the class of types that are obtained by abstraction via congruence relations on A. Then*

*(1) The natural abstraction of A is a fully abstract structure of $C_{A}$.*

*(2) Every fully abstract type of the class $C_{A}$ is isomorphic to its natural abstraction.*

**Proof.**  The required homomorphism maps the elements of the given congruence to those of the natural abstraction. For the second part, let A be fully abstract. Then there exits some congruence *R* on A/≈ such that A is isomorphic to $(A/\approx_{A})/R$. By Theorem 2, $(A/\approx_{A})/R$ must be isomorphic to A/≈.  □

Every fully abstract structure is obtained from a natural abstraction, and natural abstractions are those that insist that equality if fixed by the operations. So, fully abstract types become a candidate for a more austere characterization of the notion of abstract data type.

## 15. Semantics

While representation and abstraction are mathematically equivalent, in the sense that the notions of “homomorphism” and “congruence relation” are mathematical duals, the activities of representation and abstraction are intentionally quite different. They have different goals and starting points; what governs what is different; what is correct is different.

Buried in these remarks are two substantial philosophical differences.

One of these concerns their semantic impact. Consider the representation case. The abstract type provides a semantic interpretation for the concrete one in that it provides the correctness conditions for the concrete one: the operations of the concrete type must be in harmony with those of the abstract one. This is fleshed out by the demand that the representation function is a homomorphism. This is a minimalist requirement for the role of semantics: it must supply the conditions of correctness for the use of expressions in the language [46].

In general, a semantic account of a language of any kind must tell us when we are using an expression correctly, and when we are not. The fact that the expression means something implies that there is a whole set of normative truths about my behavior with that expression; namely, that my use of it is correct in application to certain objects and not in application to others…. The normativity of meaning turns out to be, in other words, simply a new name for the familiar fact that, regardless of whether one thinks of meaning in truth-theoretic or assertion-theoretic terms, meaningful expressions possess conditions of correct use. Kripke’s insight was to realize that this observation may be converted into a condition of adequacy on theories of the determination of meaning: any proposed candidate for the property in virtue of which an expression has meaning, must be such as to ground the ’normativity’ of meaning-it ought to be possible to read off from any alleged meaning constituting property of a word, what is the correct use of that word. [46]

A semantic account must provide us with an account of what constitutes correct use. Specifically, operations of the concrete type must be correct relative to the corresponding operations of the abstract one. In this sense the representation has a semantic function.

Abstraction principles also have semantic significance: the semantic interpretation of the functional expressions on the left hand side would appear to be given by the congruence relations on the right hand side, i.e., the latter are semantically prior to the functional expression on the left. For example, in order to understand the term ‘direction’ is to know that ‘*the direction of a*’ and ‘*the direction of b*’ refer to the same entity if and only if the lines *a* and *b* are parallel. In other words, an understanding of the concept of a direction presupposes an understanding of the concept of parallelism, but not vice-versa. This is the perspective of semantic abstractionism [17,23]. Roughly, this insists that our capacity to have singular thoughts about objects of a certain type is fixed by the truth-conditions of identity judgments about objects of that type. In the case of data types, there is a semantic role played by the concrete type together with the defined congruence relation that supplies equality for the new type. The relation is a defined relation of the concrete type: it is expressed in the language of the concrete type, the semantics of which is given. The operations of the abstracted type are then given in terms of the more concrete ones. In this sense, the abstract structure is semantically dependent upon the concrete one. Another perspective on this is provided in [35] who documents three underlying principles for any semantic theory that tie together semantics with abstraction. With its roots in Frege, the semantics of a language relies on there being a notion of reference for the terms of the language. This notion is fixed by the objects in the semantic domains. And these are fixed by the equality conditions supplied by abstraction.

While both representation and abstraction have a semantic component, the correctness conditions are reversed. For representation, it is the abstract type that provides the correctness conditions for the concrete one. For abstraction, it is the concrete type that furnishes the semantic interpretation for the abstract one.

## 16. Ontology

The most obvious difference between representation and abstraction concerns their underlying ontological assumptions. In the representational case, the construction of a homomorphism from the concrete to the abstract presupposes that both data types already exist. In contrast, in the abstraction scenario, the abstract structure is ontologically dependent upon the more concrete one: it is created from it by abstraction. Abstraction principles are taken to introduce new terms referring to sui-generis objects, and thereby provide a mechanism for the creation of new abstract types. In our example, finite sets are introduced via abstraction from finite lists. However, in the case of representation, no new objects with their types are introduced. We are given both structures ahead of time.

This ontological difference between representation and abstraction can be further illustrated by considering the the way these notions are employed in set theory [47]. We illustrate with the most fundamental of all data types, the natural numbers. Consider what might be taken as the abstract type of natural numbers.

N=<N,0,succ,rec>

where rec represents a scheme of numerical recursion. There are various representations of this structure in set theory. For example, the Von-Neumann representation of the natural numbers takes the following form.

rep:V⇒N

where

rep(ϕ)≐0rep({n,{n}})≐succ(rep(n))

and where V is the following structure.

V=<V,ϕ,succ,rec>.

ϕ is the empty set and succ(n)≐{n,{n}} etc. The Zermelo representation is only slightly different. Here succ(n)≐{n}. Indeed, in principle, any of the other possible systems of set theoretic numerals may be used to represent the natural numbers, and they all generate true arithmetical statements. But there is a significant underlying assumption here, namely that there is an independent notion of “natural number” that is being represented: representations must be representations of something. This representational scenario is at the heart of the foundations of mathematics based upon set theory. Its function is to demonstrate that all of mathematics can be “represented” in set theory.

The abstractionist approach is entirely different. First notice that representations are not intended to be definitions, and there are good reasons for not so taking them. There is an immediate concern: which one do we chose? Do we select the Von-Neumann or Zermelo numerals—or one of the other myriad of possibilities? Each of them attributes a different collection of set-theoretic, non-arithmetic, properties to numbers. For example, Zermelo and Von-Neumann differ on whether *n* is a member of n+1. While different choices attribute different sets of set-theoretic properties, from a numerical perspective, there appears to be no good reason why one account is superior to another. On the other hand, both accounts cannot both be “correct” since they contradict each other, e.g., over membership. This predicament is often called *Benacerraf’s identification problem* [48]. According to it, numbers cannot be defined as sets. Moreover, such identifications do not respect mathematical practice: we do not treat numbers as sets. Even in standard expositions of set theory, where one begins with the Von-Neumann account, this is quickly discarded, and only the arithmetical properties of the numbers employed. The non-arithmetical properties are never used in the development of mathematics inside set theory.

All this is a criticism only of the definitional perspective. From the representational one, multiple representations are not surprising, indeed they are to be welcomed since the aim of specification is to jettison or hide many of the incidental properties that give rise to such multiplicity. Generally, for abstract types, we would expect the representations to have additional properties and operations that go beyond the abstract version.

Dedekind [49] earlier claimed that set theoretic formalizations are not faithful as definitions, and links it to the need for some kind of abstraction.

The real numbers should not be identified with the corresponding cuts because those cuts have “wrong properties”; namely, as sets they contain elements, something that seems “foreign” to the real numbers themselves. Similarly, the natural numbers should not be ascribed set-theoretic or other “foreign” properties; they too should be conceived of “purely arithmetically”. If one wishes to pursue your approach I should advise not to take the class itself (the system of mutually similar systems) as the number (Anzahl, cardinal number), but rather something new (corresponding to this class), something the mind creates.

Dedekind is alluding to the creation of new things, and here abstraction comes to the fore. One interpretation of this is that numbers are to be abstracted: we need to abstract away from all the idiosyncratic properties of the various set-theoretic definitions, and just leave the numerical structure. To see how, consider an indexed family of possible numerical structures.

$$\mathrm{Num}=<Num_{i},\Delta_{i}>$$

where $\Delta_{i}=\left\{0^{i},succ^{i},rec^{i}\right\}$. These might include the Von-Neumann and Zermelo structures. All such set theoretic numeral systems are structurally isomorphic. Employing this as a congruence over the dependent sum of Num we obtain a structure:

N/≃

which is isomorphic to each of the representational systems. In particular, the Von-Neumman system satisfies the following.

V≃(V/≃)

However, the elements of (V/≃) are not sets but sui-generis objects whose totality of properties are numerical, and precisely those common to all numeral representations. In this way we abstract away from the details of individual numeral systems to obtain the new abstract structure of the natural numbers. (See [50] who employs this technique to develop a Fregean approach to Structuralism [51]. From this perspective abstract data types are “structures” and the data items themselves are “positions” in the structure [50].) Abstraction brings new sui-generis notions into existence. Not so with the representation.

## 17. Foundations

While we have employed a set-theoretic framework for our exposition, the use of set theory as the appropriate medium for the formalization of computational notions is not unproblematic. The arguments of [48] would appear to apply equally to other discrete notions: presumably, lists and finite sets are subject to the same analysis as numbers. Lists have an informal interpretation in which they have no additional set-theoretic properties but only those that pertain to list processing and manipulation. In our intuitive everyday notion of a list, lists themselves are not sets. Even within the set-theoretic regime, applying abstraction to any set-theoretic representation of a data type results in new primitive notions: the present theory of finite sets reflects a vastly different notion of “set” to that given by the Zermelo–Fraenkel axioms.

However, it is not just the data items themselves that raise issues. Feferman [52] introduces two criteria for judging the success or otherwise of the formalization of an informal mathematical notion. Let *T* be a formal theory of an informal body of mathematics *M*.

1. *T* is “adequate” for *M*, if every concept, argument, and result of *M* is represented by a (basic or defined) concept, proof, and a theorem, respectively, of *T*.
2. *T* is “faithful” to *M*, if every basic concept of *T* corresponds to a basic concept of *M* and every axiom and rule of *T* corresponds to or is implicit in the assumptions and reasoning followed in *M* (i.e., *T* does not go beyond *M* conceptually or in principle).

The Zermelo–Fraekel set theory does not provide a faithful formalization of the computer science notion of type. The primary purpose of the latter is a way of classifying the objects of the language: it acts as barrier to semantic nonsense. Furthermore, for practical reasons, type membership must be a decidable judgment: we require a type-checker to be part of the implementation of the language. While set theory is an adequate formalization of the computational notion of type, it is not faithful: it goes beyond the reasoning associated with the role of types.

This argues for a more radical approach in which the computational notion of type is taken as the fundamental one. Indeed, this is implicit in our informal development which took place against the backdrop of functional programming [14,15]. To formalize matters we require a mathematical framework in which both the syntactic and semantics aspects of functional languages can be formalized. While different functional languages, with different basic types and different type constructors, would give rise to different theories of types, Constructive type theory [53] provides a possible framework. But there are others. Feferman’s theories of operations and types [52], the theory of constructions [54,55], Typed predicate logic [9,36,56] and Turner’s theories of operations and types [57], might all be employed. Finally, Type theory and category theory are close relations. This would also be a possible framework for abstraction—some might think the natural one.

## 18. Conclusions

Abstraction and representation are two of the fundamental notions of contemporary computer science. While they are mathematically dual notions they are philosophically quite different.

While they both have semantic import, they differ in terms of what provides the semantic interpretation of what. For abstraction, the source type provides the semantic foundations for the abstracted one by supplying its equality conditions. For representation, the abstract type supplies the correctness conditions for the source type acting as a representation.

Ontologically, they differ in that representation assumes that both structures are in place, whereas abstraction brings new structures into being. Abstraction generates new types, representation enables their use in computation by supplying a more concrete representation.

These differences apply equally well to the case where one of the data types is physical. This demonstrates that the relationship between the physical and abstract types goes beyond extensional agreement: it brings an intentional aspect that is materialized by the intentions to locate a representation for one type in another or abstract a new type from an existing one—or a family of such. 

## Figures and Tables

**Figure 9:**
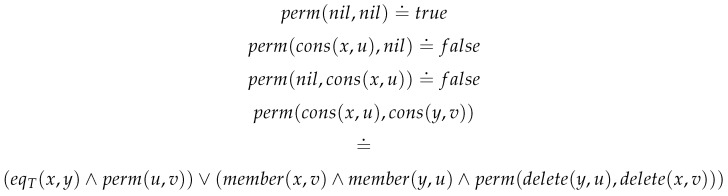
perm:L[T]⊗L[T]⇒Bool.

**Figure 10:**
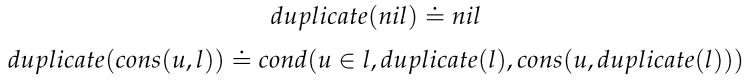
duplicate:L[T]⇒L[T].

**Figure 11:**
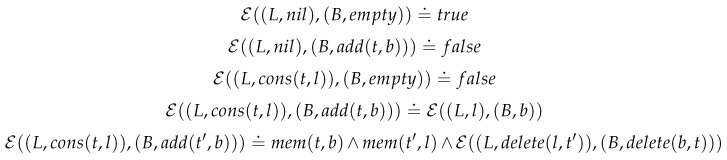
Extensional Equivalence.

## Data Availability

Data sharing not applicable.
